# Supplementary material for: A biochemical mechanism for time-encoding memory formation within individual synapses of Purkinje cells
Source: PLoS One. 2021 May 7;16(5):e0251172. doi: 10.1371/journal.pone.0251172 (PMC8104431; doi:10.1371/journal.pone.0251172)
Supplement: S1 Text — (PDF) [file pone.0251172.s001.pdf]

S1 Text. Parameter values of the Purkinje cell model

$$
\begin{aligned}
C_s &= 1.5\mu\text{F}/cm^2, C_d = 1.5\mu\text{F}/cm^2, R = 0.75, g_{Na} = 40\text{mS}/cm^2,\\
g_{Ks} &= 8.75\text{mS}/cm^2, g_{I_H} = 0.03\text{mS}/cm^2, g_{Kd(slow)} = 12\text{mS}/cm^2\\
g_{leak} &= 0.032\text{mS}/cm^2, E_{Na^+} = 45\text{mV}, E_{K^+} = -95\text{mV}, E_{I_H} = -20.0\text{mV},\\
E_{leak} &= -77\text{mV}, I_i = 0.2\mu\text{A},\\
g_{GIRK} &= 2.403\text{mS}/cm^2 \text{ (minimal model)},\\
g_{GIRK} &= 1.392\text{mS}/cm^2 \text{ (full model)}
\end{aligned}
$$

The slightly different values of g_{GIRK} for the minimal and the full model are due to the fact that the maximum value reached by the G-protein activity in the full model is slightly higher compared to the minimal model.
