# Supplementary material for: A biochemical mechanism for time-encoding memory formation within individual synapses of Purkinje cells
Source: PLoS One. 2021 May 7;16(5):e0251172. doi: 10.1371/journal.pone.0251172 (PMC8104431; doi:10.1371/journal.pone.0251172)
Supplement: S2 Text — (PDF) [file pone.0251172.s002.pdf]

S2 Text. Hill's equation

Hill's equation gives the fraction of proteins saturated by a ligand at a given concentration of the ligand in solution [1]. Since, in the case of Acetyl cyclase (AC), only one unit of G_α subunit binds to it, the Hill's coefficient will be 1. The fraction of AC bound by a G_α subunit to the total available amount of AC at a concentration $[G_\alpha]$ is given by

$$\frac{[Blocked\ AC]}{[AC]} = \frac{[G_\alpha]}{K_d + [G_\alpha]}, \tag{1}$$

where K_d is the disassociation constant of AC with a G_α subunit. However, we are interested in free AC. So, the fraction of free AC will be given by

$$\frac{[Active\ AC]}{[AC]} = 1 - \frac{[Blocked\ AC]}{[AC]} = \frac{K_d}{K_d + [G_\alpha]}. \tag{2}$$

Since the concentration of $[G_\alpha]$ is proportional to its activity, (Eq. 2) can be written as

$$\frac{[Active\ AC]}{[AC]} = \frac{K_d}{K_d + x}, \tag{3}$$

where x denotes the G-protein activity.

References

[1] Gesztelyi R, Zsuga J, Kemeny-Beke A, Varga B, Juhasz B, Tosaki A. The Hill equation and the origin of quantitative pharmacology. Archive for History of Exact Sciences. 2012;66:427–438. doi:10.1007/s00407-012-0098-5.
