# Supplementary material for: A biochemical mechanism for time-encoding memory formation within individual synapses of Purkinje cells
Source: PLoS One. 2021 May 7;16(5):e0251172. doi: 10.1371/journal.pone.0251172 (PMC8104431; doi:10.1371/journal.pone.0251172)
Supplement: S3 Text — (PDF) [file pone.0251172.s003.pdf]

S3 Text. Details of the comprehensive mathematical model

Below we provide the list of biochemical reactions that are taken into account in our comprehensive mathematical model.

In the following, R_2C_2 is inactive PKA and C is active PKA. R7 denotes the $mGluR_7$ receptor, while its subscripts correspond to the different states shown in (Fig. 2).

$$R_2C_2 + cAMP \underset{k_{r1}}{\overset{k_{f1}}{\rightleftharpoons}} R_2C_2{-}cAMP \tag{1}$$

$$R_2C_2{-}cAMP + cAMP \underset{k_{r2}}{\overset{k_{f2}}{\rightleftharpoons}} R_2C_2{-}2\,cAMP \tag{2}$$

$$R_2C_2{-}2\,cAMP + cAMP \underset{k_{r3}}{\overset{k_{f3}}{\rightleftharpoons}} R_2C_2{-}3\,cAMP \tag{3}$$

$$R_2C_2{-}3\,cAMP + cAMP \underset{k_{r4}}{\overset{k_{f4}}{\rightleftharpoons}} R_2C_2{-}4\,cAMP \tag{4}$$

$$R_2C_2{-}4\,cAMP \underset{k_{r5}}{\overset{k_{f5}}{\rightleftharpoons}} PKA + R_2C_1{-}4\,cAMP \tag{5}$$

$$R_2C_1{-}4\,cAMP \underset{k_{r6}}{\overset{k_{f6}}{\rightleftharpoons}} PKA + R_2{-}4\,cAMP \tag{6}$$

$$ATP \xrightarrow{AC} cAMP \tag{7}$$

$$cAMP \xrightarrow{PDE} AMP \tag{8}$$

$$AMP \underset{k_{r9}}{\overset{k_{f9}}{\rightleftharpoons}} ATP \tag{9}$$

$$R_{7p} \xrightarrow{Glutamate} R_{7p,a} \tag{10}$$

$$R_{7p,a} + PP_1 \underset{k_{r11}}{\overset{k_{f11}}{\rightleftharpoons}} R_{7p,a}{-}PP_1 \xrightarrow{k_{cat11}} R_{7d} + PP_1 \tag{11}$$

$$R_{7d} + PKA \underset{k_{r12}}{\overset{f(PKA)k_{f12}}{\rightleftharpoons}} R_{7d}{-}PKA \xrightarrow{k_{cat12}} R_{7p,a} + PKA \tag{12}$$

$$R_{7d} \underset{k_{r13}}{\overset{k_{f13}}{\rightleftharpoons}} R_{7b} \tag{13}$$

$$R_{7b} + PKA \underset{k_{r14}}{\overset{f(PKA)k_{f14}}{\rightleftharpoons}} R_{7b}{-}PKA \xrightarrow{k_{cat14}} R_{7p,n} + PKA \tag{14}$$

$$R_{7p,n} \xrightarrow{k_{f15}} R_{7p} \tag{15}$$

$$G_{\alpha\beta\gamma} \underset{RGS_8}{\overset{R_{7p,a}}{\rightleftharpoons}} {G_\alpha}^* + G_{\beta\gamma} \tag{16}$$

$${G_\alpha}^* + AC \rightleftharpoons {G_\alpha}^*{-}AC \tag{17}$$

In addition to the assumptions discussed in Model Conceptualization: Proposed biochemical mechanism, we make two additional assumptions to translate the biochemcial reactions into the ordinary differential equations that describe our comprehensive model:

1. As various proteins are relatively close to one other because of the AKAP scaffold protein, their rate of reaction kinetics increases significantly. Multiple studies [1, 2, 3, 4] have used a concept of effective concentration, which is obtained by multiplying a scalar factor to the actual concentration of the protein or substrate, to model the effect of AKAP on protein reaction dynamics. We introduce two scalar factors in our model to capture the accelerated kinetics of two enzymes: PDE and AC. As the conditional response is comprised of both activation and deactivation of GIRK ion channels, a scalar factor $\lambda_1 = 8.0$ is used to increase the effective concentration of the PDE enzyme to get an appropriate deactivation time-scale of the conditional response. However, an increase in the effective concentration of PDE needs to be compensated by an increase in the effective concentration of AC enzymes. This ensures a strong PKA activity, which is required to maintain phosphorylated state of the $mGluR_7$ receptor. Thus, a scalar factor $\lambda_2 = 260.0$ is used

to increase the effective concentration of AC enzymes. These two scalar factors are significantly below the expected maximum possible value of 3000 proposed elsewhere [5, 6]. The value of λ_2 is relatively high because the value of k_{cat7} associated with AC enzymes is 50 times smaller than k_{cat8} for PDE enzymes (see S1 Table), Yet, to maintain enough [cAMP] to activate PKA, these two reaction rates need to be comparable to one other, which is achieved by our choice of scalar factors for the effective concentrations.

2. PKA and PP1 both can bind to the same AKAP scaffold protein [7, 8] and interact with the same target protein, i.e., the mGluR$_7$ receptor. In such a case, PKA can potentially bind closer to the receptor or offer steric hindrance to PP1 interacting with the same receptor. Either way, the rate constants of the protein interactions are expected to be influenced. For the comprehensive model, the former case is considered where PKA binds closer to the receptor and interacts strongly when PKA activity is high, while it interacts weakly when its activity is low as PKA catalytic units are blocked by its regulatory units [9]. To account for such a behaviour of PKA, a dimensionless sigmodial dependence with exponent 3 is used which is expressed as $f(\text{PKA})$ in the chemical reaction (Eqs. 13,15),

$$f(\text{PKA}) = \frac{[\text{PKA}]^3}{C_0^3 + [\text{PKA}]^3} \tag{18}$$

Here, C_0 is a constant and equals to total [PP1]. When [PKA] is high, $k_{f12}f(\text{PKA}) >> k_{f11}$, which means that the phosphorylated state of the receptor is more favorable compared to the case when [PKA] is low, $k_{f12}f(\text{PKA}) << k_{f11}$ in which case the dephosphorylated state of the receptor is more favorable. The term $k_{f12}f(\text{PKA})$ can be considered an effective rate constant and the value of the ratio $\frac{k_{f12}f(\text{PKA})}{k_{f11}}$ decides which state of the receptor is more favorable. Hence, $f(\text{PKA})$ ensures that the receptor's state switches between a phosphorylated and a dephosphorylated state depending on the PKA activity under competitive interactions of PKA and PP1 proteins. Note that exponents lower than 3 in $f(\text{PKA})$ render a slower change in the receptor's activity, which stretches the conditional response profile, while for larger values there is no discernible difference.

Below we provide the reaction kinetic equations for the set of biochemical reactions given above under the stated assumptions.

$$\frac{d[\text{R}_2\text{C}_2]}{dt} = -k_{f1}[\text{R}_2\text{C}_2][\text{cAMP}] + k_{r1}[\text{R}_2\text{C}_2\text{c}_1] \tag{19}$$

$$\frac{d[\text{R}_2\text{C}_2\text{c}_1]}{dt} = k_{f1}[\text{R}_2\text{C}_2][\text{cAMP}] - k_{r1}[\text{R}_2\text{C}_2\text{c}_1] - (k_{f2}[\text{R}_2\text{C}_2\text{c}_1][\text{cAMP}] \tag{20}$$

$$- k_{r2}[\text{R}_2\text{C}_2\text{c}_2]) \tag{21}$$

$$\frac{d[\text{R}_2\text{C}_2\text{c}_2]}{dt} = k_{f2}[\text{R}_2\text{C}_2\text{c}_1][\text{cAMP}] - k_{r2}[\text{R}_2\text{C}_2\text{c}_2] - (k_{f3}[\text{R}_2\text{C}_2\text{c}_2][\text{cAMP}] \tag{22}$$

$$- k_{r3}[\text{R}_2\text{C}_2\text{c}_3]) \tag{23}$$

$$\frac{d[\text{R}_2\text{C}_2\text{c}_3]}{dt} = k_{f3}[\text{R}_2\text{C}_2\text{c}_2][\text{cAMP}] - k_{r3}[\text{R}_2\text{C}_2\text{c}_3] - (k_{f4}[\text{R}_2\text{C}_2\text{c}_3][\text{cAMP}] \tag{24}$$

$$- k_{r4}[\text{R}_2\text{C}_2\text{c}_4]) \tag{25}$$

$$\frac{d[\text{R}_2\text{C}_2\text{c}_4]}{dt} = k_{f4}[\text{R}_2\text{C}_2\text{c}_3][\text{cAMP}] - k_{r4}[\text{R}_2\text{C}_2\text{c}_4] \tag{26}$$

$$- (k_{f5}[\text{R}_2\text{C}_2\text{c}_4] - k_{r5}[\text{pka}][\text{R}_2\text{C}_1\text{c}_4]) \tag{27}$$

$$\frac{d[\text{R}_2\text{C}_1\text{c}_4]}{dt} = k_{f5}[\text{R}_2\text{C}_2\text{c}_4] - k_{r5}[\text{pka}][\text{R}_2\text{C}_1\text{c}_4] - (k_{f6}[\text{R}_2\text{C}_1\text{c}_4] - k_{r6}[\text{pka}][\text{R}_2\text{c}_4]) \tag{28}$$

$$\frac{d[\text{R}_2\text{c}_4]}{dt} = k_{f6}[\text{R}_2\text{C}_1\text{c}_4] - k_{r6}[\text{pka}][\text{R}_2\text{c}_4] \tag{29}$$

$$\frac{d[\text{pka}]}{dt} = k_{f5}[\text{R}_2\text{C}_2\text{c}_4] - k_{r5}[\text{pka}][\text{R}_2\text{C}_1\text{c}_4] + k_{f6}[\text{R}_2\text{C}_1\text{c}_4] - k_{r6}[\text{pka}][\text{R}_2\text{c}_4] \tag{30}$$

$$\frac{d[\text{ATP}]}{dt} = -\frac{\lambda_1 k_{cat7}[\text{AC}][\text{ATP}]}{k_{m7} + [\text{ATP}]} + k_{f9}[\text{AMP}] - k_{r9}[\text{ATP}] \tag{31}$$

$$\frac{d[\text{cAMP}]}{dt} = \frac{\lambda_1 k_{cat7}[\text{AC}][\text{ATP}]}{k_{m7} + [\text{ATP}]} - (k_{f1}[\text{R}_2\text{C}_2][\text{cAMP}] - k_{r1}[\text{R}_2\text{C}_2\text{c}_1]) \tag{32}$$
$$- (k_{f2}[\text{R}_2\text{C}_2\text{c}_1][\text{cAMP}] - k_{r2}[\text{R}_2\text{C}_2\text{c}_2]) - (k_{f3}[\text{R}_2\text{C}_2\text{c}_2][\text{cAMP}]$$
$$- k_{r3}[\text{R}_2\text{C}_2\text{c}_3]) - (k_{f4}[\text{R}_2\text{C}_2\text{c}_3][\text{cAMP}] - k_{r4}[\text{R}_2\text{C}_2\text{c}_4])$$
$$- \frac{\lambda_2 k_{cat8}[\text{PDE}][\text{cAMP}]}{(k_{m8} + c)}$$

$$\frac{d[\text{AMP}]}{dt} = \frac{\lambda_2 k_{cat8}[\text{PDE}][\text{cAMP}]}{(k_{m8} + c)} - k_{f9}[\text{AMP}] + k_{r9}[\text{ATP}] \tag{33}$$

$$\frac{d[\text{R7}_p]}{dt} = -k_{f10}[\text{I}][\text{R7}_p] + k_{f15}[\text{R7}_{p,n}] \tag{34}$$

$$\frac{d[\text{R7}_{p,a}]}{dt} = k_{f10}[\text{I}][\text{R7}_p] - k_{f11}[\text{R7}_{p,a}]([\text{PP1}] - [\text{R7}_{p,a}\text{-PP1}]) + k_{r11}[\text{R7}_{p,a}\text{-PP1}] \tag{35}$$
$$+ k_{cat12}[\text{R7}_d\text{-PKA}]$$

$$\frac{d[\text{R7}_{p,a}\text{-PP1}]}{dt} = k_{f11}[\text{R7}_{p,a}]([\text{PP1}] - [\text{R7}_{p,a}\text{-PP1}]) - (k_{r11} + k_{cat11})[\text{R7}_{p,a}\text{-PP1}] \tag{36}$$

$$\frac{d[\text{R7}_d]}{dt} = k_{cat11}[\text{R7}_{p,a}\text{-PP1}] - \frac{k_{f12}[\text{PKA}]^3}{C_0^3 + [\text{PKA}]^3}[\text{R7}_d]([\text{PKA}] - [\text{R7}_d\text{-PKA}]) \tag{37}$$
$$+ k_{r12}[\text{R7}_d\text{-PKA}] - k_{f13}[\text{R7}_d] + k_{r13}[\text{R7}_b]$$

$$\frac{d[\text{R7}_d\text{-PKA}]}{dt} = \frac{k_{f12}[\text{PKA}]^3}{C_0^3 + [\text{PKA}]^3}[\text{R7}_d]([\text{PKA}] - [\text{R7}_d\text{-PKA}]) - (k_{r12} + k_{cat12})[\text{R7}_d\text{-PKA}] \tag{38}$$

$$\frac{d[\text{R7}_b]}{dt} = k_{f13}[\text{R7}_d] - k_{r13}[\text{R7}_b] - \frac{k_{f14}[\text{PKA}]^3}{C_0^3 + [\text{PKA}]^3}[\text{R7}_d]([\text{PKA}] - [\text{R7}_b\text{-PKA}]) \tag{39}$$
$$- k_{r14}[\text{R7}_b\text{-PKA}]$$

$$\frac{d[\text{R7}_b\text{-PKA}]}{dt} = \frac{k_{f14}[\text{PKA}]^3}{C_0^3 + [\text{PKA}]^3}[\text{R7}_d]([\text{PKA}] - [\text{R7}_d\text{-PKA}]) - (k_{r14} + k_{cat14})[\text{R7}_b\text{-PKA}] \tag{40}$$

$$\frac{d[\text{R7}_{p,n}]}{dt} = k_{cat14}[\text{R7}_b\text{-PKA}] - k_{f15}[\text{R7}_{p,n}] \tag{41}$$

$$\frac{d[\text{G-protein}]}{dt} = k_{gp}([\text{R7}_{p,a}] - [\text{G-protein}]) \tag{42}$$

$$[AC](t) = \frac{K_D[AC_0]}{K_D + [\text{G-protein}]} \tag{43}$$

For each of the 14 kinetic parameters (out of 33 parameters in total) that have not been measured directly, a value is selected from within the acceptable ranges of $1M^{-1}s^{-1}$ to $10^{10}M^{-1}s^{-1}$ observed through in-vitro experiments [10, 11, 12, 13, 14, 15]. However, in in-vivo situations, the rate constants can change by a couple of order of magnitudes depending on type of protein interaction and pH or different ion concentrations [15]. This implies that some reactions can potentially occur at a rate faster than the above mentioned maximum rate measured. S1 Table summarizes the values of all rate constants used in the full model. Note that the higher values for some of the rate constant are selected to ensure that the receptor's state switches between phosphorylated or dephosphorylated states depending on PKA activity as discussed earlier. The values of the other rate constants are basically to approximate the time scale of the conditional response. The conditional response involves multiple protein interactions between the activation of the mGluR$_7$ receptor and the opening of the GIRK ion channel, which marks the onset of the conditional response. As a result, the time scale of activation of the receptor must be faster than the time scale of the fastest onset of the conditional response, which has been measured experimentally to be about $6ms$ [16]. That is why a value of $1ms$ is used for the time scale of activation of the receptor and, given that the amount of receptor is of the order of $1\mu M$, this gives a value of $1000\mu M^{-1}s^{-1}$ for the k_{f10}. In fact, we use the same time scale of $1ms$ in the minimal model for τ_2.

References

[1] Zhang J, Ma Y, Taylor SS, Tsien RY. Genetically encoded reporters of protein kinase A activity reveal impact of substrate tethering. Proceedings of the National Academy of Sciences. 2001;98(26):14997–15002. doi:10.1073/pnas.211566798.

[2] Saucerman JJ, Brunton LL, Michailova AP, McCulloch AD. Modeling β-Adrenergic Control of Cardiac Myocyte Contractility in Silico. Journal of Biological Chemistry. 2003;278(48):47997–48003. doi:10.1074/jbc.m308362200.

[3] Saucerman JJ, Healy SN, Belik ME, Puglisi JL, McCulloch AD. Proarrhythmic Consequences of a KCNQ1 AKAP-Binding Domain Mutation. Circulation Research. 2004;95(12):1216–1224. doi:10.1161/01.res.0000150055.06226.4e.

[4] Darbar D, Roden DM, Ali MF, Yang T, Wathen MS. Himalayan T Waves in the Congenital Long-QT Syndrome. Circulation. 2005;111(11):e161–e161. doi:10.1161/01.cir.0000159092.92366.cd.

[5] Zeke A, Lukács M, Lim WA, Reményi A. Scaffolds: interaction platforms for cellular signalling circuits. Trends in Cell Biology. 2009;19(8):364–374. doi:10.1016/j.tcb.2009.05.007.

[6] Schreiber G, Haran G, Zhou HX. Fundamental aspects of protein-protein association kinetics. Chemical reviews. 2009;109(3):839–60. doi:10.1021/cr800373w.

[7] Greenwald EC, Saucerman JJ. Bigger, Better, Faster: Principles and Models of AKAP Anchoring Protein Signaling. Journal of Cardiovascular Pharmacology. 2011;58:462. doi:10.1097/fjc.0b013e31822001e3.

[8] Logue JS, Scott JD. Organizing signal transduction through A-kinase anchoring proteins (AKAPs). The FEBS journal. 2010;277(21):4370–5. doi:10.1111/j.1742-4658.2010.07866.x.

[9] Søberg K, Skålhegg BS. The Molecular Basis for Specificity at the Level of the Protein Kinase a Catalytic Subunit. Frontiers in Endocrinology. 2018;9:538. doi:10.3389/fendo.2018.00538.

[10] Xie ZR, Chen J, Wu Y. Predicting Protein-protein Association Rates using Coarse-grained Simulation and Machine Learning. Scientific reports. 2017;7(1):46622. doi:10.1038/srep46622.

[11] Shen B, Hage T, Sebald W. Global and Local Determinants for the Kinetics of Interleukin-4/Interleukin-4 Receptor α Chain Interaction. European Journal of Biochemistry. 1996;240(1):252–261. doi:10.1111/j.1432-1033.1996.0252h.x.

[12] Wallis R, Leung KY, Osborne MJ, James R, Moore GR, Kleanthous C. Specificity in Protein-Protein Recognition: Conserved Im9 Residues Are the Major Determinants of Stability in the Colicin E9 DNase-Im9 Complex. Biochemistry. 1998;37(2):476–485. doi:10.1021/bi971884a.

[13] Radić Z, Kirchhoff PD, Quinn DM, McCammon JA, Taylor P. Electrostatic Influence on the Kinetics of Ligand Binding to Acetylcholinesterase. Journal of Biological Chemistry. 1997;272(37):23265–23277. doi:10.1074/jbc.272.37.23265.

[14] Schreiber G, Fersht AR. Interaction of barnase with its polypeptide inhibitor barstar studied by protein engineering. Biochemistry. 1993;32(19):5145–50.

[15] Schreiber G, Fersht AR. Rapid, electrostatically assisted association of proteins. Nature Structural Biology. 1996;3(5):427–431. doi:10.1038/nsb0596-427.

[16] Johansson F, Jirenhed DA, Rasmussen A, Zucca R, Hesslow G. Memory trace and timing mechanism localized to cerebellar Purkinje cells. Proceedings of the National Academy of Sciences. 2014;111:14930–14934. doi:10.1073/pnas.1415371111.
