# Supplementary material for: A biochemical mechanism for time-encoding memory formation within individual synapses of Purkinje cells
Source: PLoS One. 2021 May 7;16(5):e0251172. doi: 10.1371/journal.pone.0251172 (PMC8104431; doi:10.1371/journal.pone.0251172)
Supplement: S1 Table — (PDF) [file pone.0251172.s004.pdf]

S1 Table. Values of kinetic parameters used in the comprehensive mathematical model

Parameter	Actual value	Units	References
k_{f1}	54.0	$\mu M^{-1}s^{-1}$	[1]
k_{r1}	33.0	s^{-1}	[1]
k_{f2}	54.0	$\mu M^{-1}s^{-1}$	[1]
k_{r2}	33.0	s^{-1}	[1]
k_{f3}	75.0	$\mu M^{-1}s^{-1}$	[1]
k_{r3}	110.0	s^{-1}	[1]
k_{f4}	75.0	$\mu M^{-1}s^{-1}$	[1]
k_{r4}	110.0	s^{-1}	[1]
k_{f5}	60.0	s^{-1}	[1]
k_{r5}	18.0	$\mu M^{-1}s^{-1}$	[1]
k_{f6}	60.0	s^{-1}	[1]
k_{r6}	18.0	$\mu M^{-1}s^{-1}$	[1]
k_{cat7}	0.2	s^{-1}	[2]
k_{m7}	1030.0	μM	[2]
k_{cat8}	10.0	s^{-1}	[1, 3, 4, 5, 6, 7]
k_{m8}	1.8	μM	[1, 3, 4, 5, 6, 7]
k_{f9}	100.0	s^{-1}	[8]
k_{r9}	1.0	s^{-1}	[8]
k_{f10}	1.0e3	$\mu M^{-1}s^{-1}$	Within acceptable range [9]
k_{f11}	1.0e2	$\mu M^{-1}s^{-1}$	Within acceptable range [9]
k_{r11}	1.0e4	s^{-1}	Within acceptable range [9]
k_{cat11}	1.0e3	s^{-1}	Within acceptable range [9]
k_{f12}	1.0e4	$\mu M^{-1}s^{-1}$	Within acceptable range [9]
k_{r12}	1.0e2	s^{-1}	Within acceptable range [9]
k_{cat12}	1.0e4	s^{-1}	Within acceptable range [9]
k_{f13}	1.0e2	s^{-1}	Within acceptable range [9]
k_{r13}	1.0e1	s^{-1}	Within acceptable range [9]
k_{f14}	1.0e4	$\mu M^{-1}s^{-1}$	Within acceptable range [9]
k_{r14}	1.0e2	s^{-1}	Within acceptable range [9]
k_{cat14}	1.0e4	s^{-1}	Within acceptable range [9]
k_{f15}	0.01	s^{-1}	Within acceptable range [9]
k_{gp}	21.0	s^{-1}	Within acceptable range [9]
K_D	0.08	μM	Obtained by taking the ratio of k_r and k_f value given in [10] for the binding between AC and G_α unit.

References

[1] Hayer A, Bhalla US. Molecular Switches at the Synapse Emerge from Receptor and Kinase Traffic. PLoS Computational Biology. 2005;1(2):e20. doi:10.1371/journal.pcbi.0010020.

[2] Neves SR, Tsokas P, Sarkar A, Grace EA, Rangamani P, Taubenfeld SM, et al. Cell shape and negative links in regulatory motifs together control spatial information flow in signaling networks. Cell. 2008;133(4):666–80. doi:10.1016/j.cell.2008.04.025.

[3] Hoffmann R, Baillie GS, MacKenzie SJ, Yarwood SJ, Houslay MD. The MAP kinase ERK2 inhibits the cyclic AMP-specific phosphodiesterase HSPDE4D3 by phosphorylating it at Ser579. The EMBO Journal. 1999;18(4):893–903. doi:10.1093/emboj/18.4.893.

[4] Bender AT, Beavo JA. Cyclic Nucleotide Phosphodiesterases: Molecular Regulation to Clinical Use. Pharmacological Reviews. 2006;58(3):488–520. doi:10.1124/pr.58.3.5.

[5] Salanova M, Jin SLC, Conti M. Heterologous Expression and Purification of Recombinant Rolipram-Sensitive Cyclic AMP-Specific Phosphodiesterases. Methods. 1998;14(1):55–64. doi:10.1006/meth.1997.0565.

[6] Wang P, Myers JG, Wu P, Cheewatrakoolpong B, Egan RW, Billah MM. Expression, Purification, and Characterization of Human cAMP-Specific Phosphodiesterase (PDE4) Subtypes A, B, C, and D. Biochemical and Biophysical Research Communications. 1997;234(2):320–324. doi:10.1006/bbrc.1997.6636.

[7] Huston E, Lumb S, Russell A, Catterall C, Ross HA, Steele RM, et al. Molecular cloning and transient expression in COS7 cells of a novel human PDE4B cAMP-specific phosphodiesterase, HSPDE4B3. Biochemical Journal. 1997;328(2):549–558. doi:10.1042/bj3280549.

[8] Hardie DG. Keeping the home fires burning: AMP-activated protein kinase. Journal of The Royal Society Interface. 2018;15(138):20170774. doi:10.1098/rsif.2017.0774.

[9] Xie ZR, Chen J, Wu Y. Predicting Protein-protein Association Rates using Coarse-grained Simulation and Machine Learning. Scientific reports. 2017;7(1):46622. doi:10.1038/srep46622.

[10] Song RS, Massenburg B, Wenderski W, Jayaraman V, Thompson L, Neves SR. ERK regulation of phosphodiesterase 4 enhances dopamine-stimulated AMPA receptor membrane insertion. Proceedings of the National Academy of Sciences of the United States of America. 2013;110(38):15437–42. doi:10.1073/pnas.1311783110.
