# Supplementary material for: A biochemical mechanism for time-encoding memory formation within individual synapses of Purkinje cells
Source: PLoS One. 2021 May 7;16(5):e0251172. doi: 10.1371/journal.pone.0251172 (PMC8104431; doi:10.1371/journal.pone.0251172)
Supplement: S2 Table — (PDF) [file pone.0251172.s005.pdf]

S2 Table. (Initial) Concentration of various biomolecules for comprehensive mathematical model

As most biomolecules are associated with the protein complex comprised of the GIRK ion channel, the initial concentrations of various biomolecules are determined relative to the GIRK ion channel abundance. In such a case, a typical value of 100 ion channels per synatic bouton [1] is assumed as there is no experimentally measured value for the total number of GIRK ion channels at the Purkinje cell's synapses. The typical area of the synaptic bouton is about $2\mu m^2$ [2], which gives a surface molecular density of approximately $0.5 nmoles/m^2$ or roughly 1μM by volume if the synaptic bouton is approximate by a sphere with a radius of roughly 1μm. From this amount of GIRK ion channels, one can determine the amounts of $mGluR_7$ receptors, G-proteins, PKA, PP1, AC and PDE enzymes as discussed in Model Conceptualization: Proposed biochemical mechanism. Similarly, one can estimate [Glutamate]$\approx 1\mu M$ under the assumption that at least 100 molecules of glutamate must be present at a synaptic bouton to activate 100 ion channels present at the postsynaptic membrane. Based on these assumptions, the table below shows the specific initial concentrations of various biomolecules used in the model.

**Protein**	**Initial concentration**	**References**
GIRK ion channel	$1\mu M$	[1, 2, 3]
$mGluR_7$	$4\mu M$	[1, 2, 3]
G-protein	$4\mu M$	[1, 2, 3]
R2C2 i.e., (PKA)	$\sim 4\mu M$	As PKA binds to AKAP protein [4, 5, 6, 1, 2, 3]
PP1	$\sim 4\mu M$	As PP1 binds to AKAP protein as well [4, 5, 6, 1, 2, 3]
AC	$\sim 2\mu M$	As AC also binds to AKAP protein [7]
ATP	2000mM	[7]
PDE	$1.25\mu M$	[PDE] can vary between 0 and $4\mu M$ depending on how many molecules of PDE bind to each TEC unit.
I	$1.0\mu M$	I signifies [Glutamate], whose value for when the conditional stimulus is on is determined as discussed above using [1, 2].

For the remaining intermediate biomolecule species, we set them to 0 initially.

References

[1] Patrizio A, Specht CG. Counting numbers of synaptic proteins: absolute quantification and single molecule imaging techniques. Neurophotonics. 2016;3(4):041805. doi:10.1117/1.nph.3.4.041805.

[2] Knodel MM, Geiger R, Ge L, Bucher D, Grillo A, Wittum G, et al. Synaptic bouton properties are tuned to best fit the prevailing firing pattern. Frontiers in computational neuroscience. 2014;8:101. doi:10.3389/fncom.2014.00101.

[3] Sadja R, Alagem N, Reuveny E. Gating of GIRK Channels Details of an Intricate, Membrane-Delimited Signaling Complex. Neuron. 2003;39:9–12. doi:10.1016/s0896-6273(03)00402-1.

[4] Davare MA, Avdonin V, Hall DD, Peden EM, Burette A, Weinberg RJ, et al. A beta 2 Adrenergic Receptor Signaling Complex Assembled with the Ca2+ Channel Cav1.2. Science. 2001;293(5527):98–101. doi:10.1126/science.293.5527.98.

[5] Lavine N, Ethier N, Oak JN, Pei L, Liu F, Trieu P, et al. G Protein-coupled Receptors Form Stable Complexes with Inwardly Rectifying Potassium Channels and Adenylyl Cyclase. Journal of Biological Chemistry. 2002;277(48):46010–46019. doi:10.1074/jbc.m205035200.

[6] Westphal RS, Tavalin SJ, Lin JW, Alto NM, Fraser IDC, Langeberg LK, et al. Regulation of NMDA Receptors by an Associated Phosphatase-Kinase Signaling Complex. Science. 1999;285(5424):93–96. doi:10.1126/science.285.5424.93.

[7] Song RS, Massenburg B, Wenderski W, Jayaraman V, Thompson L, Neves SR. ERK regulation of phosphodiesterase 4 enhances dopamine-stimulated AMPA receptor membrane insertion. Proceedings of the National Academy of Sciences of the United States of America. 2013;110(38):15437–42. doi:10.1073/pnas.1311783110.
