# Supplementary material for: A biochemical mechanism for time-encoding memory formation within individual synapses of Purkinje cells
Source: PLoS One. 2021 May 7;16(5):e0251172. doi: 10.1371/journal.pone.0251172 (PMC8104431; doi:10.1371/journal.pone.0251172)
Supplement: S3 Table — (PDF) [file pone.0251172.s006.pdf]

S3 Table. Parameter sensitivity analysis of the comprehensive mathematical model

Parameter	Maximum fold change of original value	Description
k_{cat7}	2.0	Constrained by experimentally measured value [1]
[PDE]	2.0	Constrained within the expected range of values, see S2 Table for more details.
k_{cat8}	2.0	Constrained by experimentally measured value [2, 3, 4, 5, 6, 7]
k_{f11}	3.0	Parameter value within expected range of rate constants [8].
k_{gp}	3.0	Parameter value within expected range of rate constants [8].
k_{m7}	5.0	Constrained by experimentally measured value [1]
k_{m8}	5.0	Constrained by experimentally measured value [2, 3, 4, 5, 6, 7]
k_{f12}	5.0	Parameter value within expected range of rate constants [8].
k_{f13}	5.0	Parameter value within expected range of rate constants [8].
k_{f5}	10.0	Constrained by experimentally measured value [2]
k_{r5}	10.0	Constrained by experimentally measured value [2]
k_{cat11}	10.0	Parameter value within expected range of rate constants [8].
k_{f4}	20.0	Constrained by experimentally measured value [2]
k_{r4}	20.0	Constrained by experimentally measured value [2]

References

[1] Neves SR, Tsokas P, Sarkar A, Grace EA, Rangamani P, Taubenfeld SM, et al. Cell shape and negative links in regulatory motifs together control spatial information flow in signaling networks. Cell. 2008;133(4):666–80. doi:10.1016/j.cell.2008.04.025.

[2] Hayer A, Bhalla US. Molecular Switches at the Synapse Emerge from Receptor and Kinase Traffic. PLoS Computational Biology. 2005;1(2):e20. doi:10.1371/journal.pcbi.0010020.

[3] Hoffmann R, Baillie GS, MacKenzie SJ, Yarwood SJ, Houslay MD. The MAP kinase ERK2 inhibits the cyclic AMP-specific phosphodiesterase HSPDE4D3 by phosphorylating it at Ser579. The EMBO Journal. 1999;18(4):893–903. doi:10.1093/emboj/18.4.893.

[4] Bender AT, Beavo JA. Cyclic Nucleotide Phosphodiesterases: Molecular Regulation to Clinical Use. Pharmacological Reviews. 2006;58(3):488–520. doi:10.1124/pr.58.3.5.

[5] Salanova M, Jin SLC, Conti M. Heterologous Expression and Purification of Recombinant Rolipram-Sensitive Cyclic AMP-Specific Phosphodiesterases. Methods. 1998;14(1):55–64. doi:10.1006/meth.1997.0565.

[6] Wang P, Myers JG, Wu P, Cheewatrakoolpong B, Egan RW, Billah MM. Expression, Purification, and Characterization of Human cAMP-Specific Phosphodiesterase (PDE4) Subtypes A, B, C, and D. Biochemical and Biophysical Research Communications. 1997;234(2):320–324. doi:10.1006/bbrc.1997.6636.

[7] Huston E, Lumb S, Russell A, Catterall C, Ross HA, Steele RM, et al. Molecular cloning and transient expression in COS7 cells of a novel human PDE4B cAMP-specific phosphodiesterase, HSPDE4B3. Biochemical Journal. 1997;328(2):549–558. doi:10.1042/bj3280549.

[8] Xie ZR, Chen J, Wu Y. Predicting Protein-protein Association Rates using Coarse-grained Simulation and Machine Learning. Scientific reports. 2017;7(1):46622. doi:10.1038/srep46622.
