# Supplementary material for: A biochemical mechanism for time-encoding memory formation within individual synapses of Purkinje cells
Source: PLoS One. 2021 May 7;16(5):e0251172. doi: 10.1371/journal.pone.0251172 (PMC8104431; doi:10.1371/journal.pone.0251172)
Supplement: S4 Table — (PDF) [file pone.0251172.s007.pdf]

S4 Table. Parameters values for the modified G-protein subunit dynamics

**ISI (ms)**	**Minimal Model**		**Comprehensive model**	
	β	τ_3 **(ms)**	**[PDE] in** μ**M**	**1/k**$_{gp}$ **in (ms)**
200.0	8.5	60.0	1.3	50.0
300.0	6.1	96.0	1.0	66.66
400.0	4.8	140.0	0.85	100.0

As h_{GIRK} is a function of G-protein activity, the value of h_{GIRK} will be different from h^4_{GIRK}. As a result, we need to adjust the g_{GIRK} value to get similar drop in firing rate. Adjusted values of g_{GIRK} are as follows: For the minimal model $g_{GIRK} = 1.08\text{mS}/cm^2$ while for the comprehensive model $g_{GIRK} = 0.928\text{mS}/cm^2$.
