# Supplementary material for: A biochemical mechanism for time-encoding memory formation within individual synapses of Purkinje cells
Source: PLoS One. 2021 May 7;16(5):e0251172. doi: 10.1371/journal.pone.0251172 (PMC8104431; doi:10.1371/journal.pone.0251172)
Supplement: Attachment — Submitted filename: Response_to_Reviewers.pdf [file pone.0251172.s008.pdf]

Response to Reviewers:

PONE-D-19-23452
A biochemical mechanism for time-encoding memory formation within individual synapses of Purkinje cells
PLOS ONE

Dear Dr. Cymbalyuk,

Thank you for forwarding the Reviewers’ responses together with your feedback. Due to unforeseen delays including the COVID-19 pandemic, our revisions took much longer than expected and we apologize for this delay. Below we provide a detailed reply to all points raised by you and the Reviewers. Given the positive feedback from PLOS ONE before, we are confident that our manuscript now fully meets PLOS ONE’s publication criteria.

Best regards,

A. Mandwal, J. Orlandi, C. Simon, J. Davidsen.

Academic Editor:

Both reviewers find the article interesting, but request a serious revision to clarify the presentation.
The manuscript requires significant revision for clarity, the choice of the model and its parameters should be thoroughly justified and explained.

Please, consider shortening of the manuscript. For example, the "Biochemical Description" section could be eliminated. Its information could be condensed and moved partly to the intro, but mostly to the discussion. Also, there is an emphasis on describing the biochemical pathways in detail; however, the model is quite abstract.

Please, address the issue with the use of cubic differential equations which suggests that the system is sensitive to sudden changes in concentrations.

Why is the model so insensitive to coefficient variations: "Specifically, changes in the size of up to 100% of the values we use in the different figures will render above mentioned two features unchanged." What are the steady states of Eqs. 2-6?

Fix the diagram of Figure 1: Climbing fiber synapses do not take place on the side of spines.

Consider providing plots of the individual components to explain what is written on pages 28-29.

Consider, providing a table that describes the mechanism assumed in the model, such that auto-phosphorylation is modeled as a quadratic interaction.
It is not clear how the parameters of these equations were determined. How sensitive are the results to changes (absolute and relative) of these parameters?

Why is that I in equation 3 is considered linear, while this definitely saturates?

Address the issue with the assumption that tau1 changes under different conditions. Changing the time constant skips over the intrinsic dynamics of the set of the differential equation. This basically changes the window of activation of the entire biochemical network. Could you please clarify this point?

Please, fix a few typos in the Materials and Method section. For example, in eq. (7) g_K should be g_{Ks}; in Eq. (1) the derivative is for the inactivation variable “ih” shown in Eq. (7) and not for “I_h”, i.e. d(ih)/dt = … and not d(I_h)/dt =…; in Eq. (11) the time constant should be for the variable n_d (not for ih).

Please, consider in your discussion following articles (1) Galit et al, Mutual action by Gγ and Gβ for optimal activation of GIRK channels in a channel subunit-specific manner, Scientific Reports 9, Article number: 508 (2019), and (2) Pellegrino et al, GIRK channels modulate Purkinje cell excitability and synaptic transmission in mice cerebellum, Cellular Neuroscience Archive (DOI=10.3389/conf.fncel.2017.37.00008).

REPLY: As we described in more detail below in our response to the Reviewers, we have addressed all issues raised by them and echoed by you above. In particular, we have significantly revised our manuscript to clarify the presentation and also vastly extended our analysis of the proposed biochemical mechanism by proposing a full biochemical model in addition to the more abstract and minimal three-variable model. We show explicitly that our findings are robust across a large range of parameters and across both models. To this end, we also provide a detailed phase space and steady state analysis of the three-variable model, which is not possible in the full biochemical model.

Reviewer #1: The manuscript PONE-D-19-23452 “A biochemical mechanism for time-encoding memory formation within individual synapses of Purkinje cells” by Ayush Mandwal, Javier Orlandi, Christoph Simon, and Jörn Davidsen describe a biochemical mechanism that could explain some synaptic-level plasticity (memory) effect.
The manuscript has been available at doi: https://doi.org/10.1101/596148 (https://www.biorxiv.org/content/10.1101/596148v1).

This manuscript is built on two different models pieced together:
1) the biochemical model of synaptic timing build around the GIRK (G-protein coupled

Inward Rectifier Potassium) ion channels. This mechanism that has been extensively studied in the doctoral dissertation of Fredrik Johansson, Division for Neuroscience, Department of Experimental Medical Science, Lund University (Johansson F. A Purkinje cell Timing Mechanism. On the Physical Basis of a Temporal Duration Memory. Lund University; 2015) and some of the publications coming out of their research group.

In his doctoral dissertation, Johansson mentioned among others:
"That an individual neuron can learn temporal relationships suggests the existence of intracellular temporal duration memory. We demonstrate that this Purkinje cell memory is triggered by the metabotropic glutamate receptor 7 (paper II) and that the timed voltage response in large part is produced by the G-protein activated K+ channel family Kir3/GIRK (paper III)."

2) The model of the Purkinje cell of Fernandez et al, Journal of Neurophysiology 98: 278-294 (2007). However, there are a few typos in the Materials and Method section. For example, in eq. (7) g_K should be g_{Ks}; in Eq. (1) the derivative is for the inactivation variable “ih” shown in Eq. (7) and not for “I_h”, i.e. d(ih)/dt = … and not d(I_h)/dt =…; in Eq. (11) the time constant should be for the variable n_d (not for ih).

REPLY 1: Thank you for pointing out these typos, we apologize for them. We have fixed them now.

Time-Encoding protein Complexes (TEC) - the key concept and novelty of this manuscript - has some similarities to the idea introduced by (1) Galit et al, Mutual action by Gγ and Gβ for optimal activation of GIRK channels in a channel subunit-specific manner, Scientific Reports 9, Article number: 508 (2019)

REPLY 2: In the above reference paper, the authors discuss the influence of Gγ and Gβ subunits on basal and evoked currents of GIRK ion channels. Their results show that the GIRK ion channel gating can be influenced by Gγ units in the presence of Gβ units, though typically Gγ units are under regulation from G-Protein Coupled Receptors for instance mGluR7 receptors. We considered the role of the receptor behind GIRK ion channel gating dynamics in our proposed mechanism but we did not consider the role of individual G-protein units since the GIRK ion channel has a much slower time-scale for activation and deactivation compared to what is observed in the conditional response. We have now added a brief discussion of the work by Galit Tabak et al. to our manuscript [Ref. 99 in the manuscript].

and (2) Pellegrino et al, GIRK channels modulate Purkinje cell excitability and synaptic transmission in mice cerebellum, Cellular Neuroscience Archive (DOI=10.3389/conf.fncel.2017.37.00008).

REPLY 3: The above reference addresses the influence of selective activation of GIRK1 by agonist ML-297 (ML) on parallel fiber-Purkinje cell (pf-PC) synapses. Their results show activation of GIRK ion channels block the expression of Long-term Depression (LTD) while promote Long-term Potentiation (LTP) of PC-pf synapses suggesting GIRK ion channel activation involvement behind synaptic plasticity. However, increase or decrease in

synaptic strength during LTP or LTD is different from synaptic plasticity involved in the conditional response of PC. The conditional response involves *temporary* suppression of the firing rate caused by the activation of GIRK ion channel, which if translated into terms of synaptic strength would mean temporarily weakening of the synaptic strength. Thus, our proposed mechanism behind conditional response is not affected by the involvement of the GIRK ion channel in LTP or LTD of pf-PC synapse. We now cite the work by Pellegrino Lippiello et al. and added a corresponding brief discussion [Ref. 53 in the manuscript].

The key equations of the proposed synaptic-level biochemical mechanism TEC are given in Mathematical Model section (Eqs. 2-6).

My main concern is that while the authors provided some numerical results, they did not discuss at all the properties of the model in the sense of bifurcation analysis. They showed the behavior of the model (Figs. 4-8) for a set of parameters. The only paragraph where they discuss how the parameters were selected is from lines 463-471. Why is the model so insensitive to coefficient variations: "Specifically, changes in size of up to 100% of the values we use in the different figures will render above mentioned two features unchanged." What are the steady states of Eqs. 2-6? What is the stability domain of the solutions? Is there any bifurcation point? What kind? What do the bifurcation points mean for the behavior of the model?

REPLY 4: We have now added a phase space analysis based on nullclines as well as a linear stability analysis including bifurcation plots, which allow us to show and discuss different potential behaviours of our model including the fixed points and steady states of our model. Since we have been able to further reduce the dimensionality of our model to three variables (see our **Reply 14** to Reviewer #2), one can now easily identify the different bifurcations and the stability domain of the relevant fixed point. In particular, this allows us to provide specific ranges of different parameters values to establish the robustness of the proposed mechanism in the context of our three-variable model. We discuss these aspects now in great detail in our manuscript.

Moreover, we now propose a full biochemical model as well (see Materials and Method section). All parameter values for the full model are either obtained or constrained based on existing literature/experimental observations. In the three-variable model, most of the parameters directly match the ones in the full biochemical model. We set the values of the other parameters to mimic the dynamics of the full biochemical model. Indeed, the three-variable model can reproduce all main features of the full model qualitatively despite the much smaller number of parameters and variables. For the full biochemical model, we also performed a sensitivity analysis. Most of the parameters can be varied by a factor of 50 of their original values without preventing the conditional response behaviour. However, there are four parameters, which can only be varied by a factor of 2 or 3 of their original value without leading to significant qualitative changes in the conditional response behaviour of the full model. Among these four parameters is k_gp, which regulates the rate of G-protein activation and deactivation. It is also a parameter

that controls the conditional response behaviour in terms of different ISI durations. More details about the sensitivity of the various parameters is given in the manuscript.

Reviewer #2: The objective of this paper is to propose a plastic intrinsic excitability mechanism that can be used to learn the time between a conditional and unconditional stimulus in Purkinje cells. The authors propose that a link between mGluRs and GIRK channels can account for reductions of the firing rate without the need an explicit AMPA-mediated synaptic plasticity mechanism. This is an interesting proposal that would be of interest to many readers. However, the manuscript requires significant improvements to simplify the presentation, justify the parameters used, and clarify the methodology.

The manuscript is too long. It should significantly be shortened. The "Biochemical Description" section should be eliminated. Its information should be condensed and moved partly to the intro, but mostly to the discussion. Also, there is an emphasis on describing the biochemical pathways in detail; however, the model is quite abstract.

REPLY 5: While we agree with the Reviewer that the section “Biochemical Description” was too long (and we have shortened it now) we would like to take the opportunity the stress that the proposed biochemical mechanism for time-encoding memory formation is the main contribution of our work and directly connects with previous experiments and motivates new experiments. That is why the section “Biochemical Description” plays a key role in our paper. To stress this further, we have now developed a full biochemical model that includes the biochemical pathways in detail. As we show in the revised version of our manuscript, this full model is capable of generating the experimentally observed behavior using parameter values based on or constrained by existing literature. However, it is not easily amenable to a mathematical stability and bifurcation analysis. Our (previous) more abstract model – which we now have further reduced to a three-variable model (see also our **Reply 14**) and which can reproduce all main features of the full model qualitatively – on the other hand is. That’s why we discuss it in more detail in the main part of the manuscript and present the full biochemical model in detail in the Materials and Method section.

There is a lack of analysis of the robustness of the results to changes in control parameters.

REPLY 6: For the full biochemical model, we have now performed a sensitivity analysis and discuss its robustness in detail in the Materials and Method section of our revised manuscript, see also our **Reply 4** to Reviewer #1. In particular, the conditional response is robust against variations in most of the parameters. For the three-variable model, we have now identified the stability domain of the relevant fixed point among other things. We discuss these aspects in detail in our manuscript.

The use of cubic differential equations would suggest that the system is sensitive to sudden changes in concentrations.

REPLY 7: While this is often true, for our three-variable model the corresponding nullcline in v (signifying mGluR7 receptor activity) separates into v=0 and a quadratic form in the absence of a stimulus as now shown in Fig.5 . For the relevant parameter values (the dimensional ones consistent with experimental measurements and the non-dimensional ones reproducing the dynamics of the full biochemical model), there is only a single stable fixed point. This is also true in the presence of a stimulus, for which the cubic relation is in particular responsible for the conditional response behaviour.

The diagram of Figure 1 is not correct. Climbing fiber synapses do not take place on the side of spines.

REPLY 8: Thanks for pointing this out. We have corrected our mistake.

Perhaps the authors should present the mathematical model first, and then provide the evidence to use such a structure. This would be much easier to read, and would focus the manuscript on facts of importance for the simulations.

REPLY 9: Please see our **Reply 5** above.

I think the readers would benefit of plots of the individual components to explain what is written in pages 28-29.

REPLY 10: Unfortunately, we are not sure which part of our manuscript the reviewer refers to since there is no page 29 and page 28 only contains references in our version of the manuscript.

I think there should be a table that describes the mechanism assumed in the model, such that auto-phosphorylation is modeled as a quadratic interaction.

REPLY 11: We agree that this is helpful and we have now included such a table in the Materials and Method section.

It is not clear how the parameters of these equations were determined. How sensitive are the results to changes (absolute and relative) of these parameters?

REPLY 12: Please see our **Reply 4** to Reviewer #1 and **Reply 6** above.

Why is that I in equation 3 is considered linear, while this definitely saturates?

REPLY 13: In our model, I is the rate of activation of the receptor, which is determined by the stimulus but not necessarily proportional to it such that it is treated as a free parameter here. In a real scenario I might have complex spatio-temporal dynamics and depend on several external parameters, like number of released vesicles, their size, etc. For simplicity, and akin to most electrophysiological protocols, we focus only on the response of our model to constant currents.

I have an issue with the assumption that tau1 changes under different conditions. Changing the time constant skips over the intrinsic dynamics of the set of differential equation. This basically changes the window of activation of the entire biochemical network. Could you please clarify this point?

REPLY 14: Given the observation that the dynamics of proteins attached to a protein complex are quite different compared to when they are in solution, we have now concluded that, based on [3,4,5], PP1 does not need any regulation. This means that it can remain active and dephosphorylating its target protein i.e., mGluR7 receptor. However, because PKA protein also targets the same protein, its effect of phosphorylation of the target protein can overcome the effect of PP1 attached in the vicinity. Thus, in our full biochemical model, we assume PP1 to be constant. This assumption automatically reduces the dimensionality of our original (more abstract) model as well, which becomes now a three-variable model. In this three-variable model, tau1 is fixed. Instead, the value of the variable beta changes under different conditions, which corresponds to changes in PDE enzyme concentration in the full biochemical model. In particular, we obtain different conditional responses (in terms of ISI) by varying the parameters beta and tau3 simultaneously in our 3-variable model. In particular, tau3 in the 3-variable model corresponds to k_gp in the full model, which regulates activation and deactivation rates of the G-protein. This is discussed in detail in our revised manuscript.

[3] Lavine N, Ethier N, Oak JN, Pei L, Liu F, Trieu P, et al. G Protein-coupled Receptors Form Stable Complexes with Inwardly Rectifying Potassium Channels and Adenylyl Cyclase. Journal of Biological Chemistry. 2002;277(48):46010–46019.doi:10.1074/jbc.m205035200.

[4] Westphal RS, Tavalin SJ, Lin JW, Alto NM, Fraser IDC, Langeberg LK, et al. Regulation of NMDA Receptors by an Associated Phosphatase-Kinase Signaling Complex. Science. 1999;285(5424):93–96. doi:10.1126/science.285.5424.93.

[5] Bauman AL, Scott JD. Kinase- and phosphatase-anchoring proteins: harnessing the dynamic duo. Nature Cell Biology. 2002;4(8):E203–E206. doi:10.1038/ncb0802-e203.

In summary, I like the idea of the paper; however, I think it requires the authors to significantly rewrite it to simplify the presentation and elaborate on the properties of the model.
