# Supplementary material for: A biochemical mechanism for time-encoding memory formation within individual synapses of Purkinje cells
Source: PLoS One. 2021 May 7;16(5):e0251172. doi: 10.1371/journal.pone.0251172 (PMC8104431; doi:10.1371/journal.pone.0251172)
Supplement: Attachment — Submitted filename: Reviewer_comments.pdf [file pone.0251172.s009.pdf]

The authors describe a novel model of associative learning based on biochemical mechanisms driving Purkinje cell intrinsic synaptic changes. The work is interesting for both neuroscientists investigating the cerebellum and computational neuroscientists modelling cerebellum-driven behaviors.

An extensive description of their model conceptualization and implementation is provided and conclusions are solid. However, some major changes would help to improve the manuscript significantly, better supporting the conclusions and strengthening the impact it could have on cerebellum learning modelling. Those are explained more in detail below.

Conceptual comments:

- the authors should comment the applicability of their model to delay and trace conditioning. Is the model in the paper applicable to both cases? In case of delay conditioning, stimuli should co-terminate, but this does not seem the case of the described model where the CS could end after the US (e.g. the black bar in Figure 4 representing CS ends after the green bar representing the ISI. Or did I misunderstand the meaning of bars in the figure?).
- The role of repeated CS-US paired trials is not addressed in the model, as all figures report single-trial analysis. Is your model able to capture this aspect of learning (e.g. start of learning, middle of learning)? Or only after-learning responses could be simulated?
- Model parameters: all of them are derived from experiments in literature or from other literature modelling studies? None of them required any tuning/fitting procedure? This should be clarified.
- Being able to reproduce more detailed mechanisms and fit models to experimental data is not always a good reason to build detailed models: a part from this ("Yet, some of the parameters in our minimal model are effective parameters and, hence, cannot be connected to experimentally accessible parameters and molecular interactions."), could you discuss more reasons and application examples where the detailed model should be preferred with respect to the minimal one?
- The role of feedforward inhibition from Molecular Layer Interneurons is mentioned in the Introduction but not addressed in the Discussion: this should also be commented as another mechanism contributing to associative learning as reported in [Ten Brinke et al., Cell Reports, 2015] and [Boele et al., Science Adv, 2018].

The paper needs an extensive reorganization to be more concise and clear:

- The Materials and Methods section clearly describing the contribution of the authors with respect to state of the art reported in the Introduction (including the design of the biochemical model and the minimal model). The section "Biochemical description" could be summarized, for example using a table to report experimental evidence and corresponding hypotheses made by authors; alternatively, a block diagram integrating the flow diagram of Figure 2 could help to make this part more clear and concise.
- For each step/analysis presented in "Materials and Methods" (model conceptualization, model implementation in minimal and full versions, stability and sensitivity analysis methods, parametrization of the model, simulation and simulation analysis), the Results should be properly described in a corresponding section within "Results", avoiding duplication in different parts of the paper.

- some sections (e.g. Hill's model, tables with parameter values, kinetic equations of the comprehensive model – leaving only one example one in the main text) could go to Supplementary Material.
- Figure 2 and 3 are redundant.
- Table 2 should be summarized and being complementary to the text, while some concepts are repeated.
- The stability analysis should be more concise (a summary table could help)
- Figures reporting the same results for the minimal and full model (e.g. 4 and 12) could be merged together in single figures with multiple panels, reorganizing the results so that the observations common to both models are presented together.
- Model comparison and model limitations should go in Results or Discussion sections.

Clarification comments:

- Figure 4: legend covers plot; green bar not explained
- Figure 6: where is the impact of γ values?
- How was Equ. 1 derived? From literature?
- Which software was used for simulations? Is code available? If yes, link to repository should be added in the manuscript (data availability is not applicable, but code should be made available)
- Figure 13: the short stimulus is not tested? (As in Figure 7; while in Figure 7 ISI = 400ms is not tested). Those two figures should be completed. Have those modelling results been validated against data (e.g. same % decrease of Purkinje cell firing rate obtained in the model with respect to experiments?)

Minor comments:

l. 26-31: it should be specified that the Conditioned Response is generated after repeated paired presentations of the two stimuli

l. 34: the projection to cerebellar nuclei should be mentioned in describing how PC activity change controls ocular muscles.

l. 122: "the " before learning should be removed

l. 351: "=λ" ?

l. 642: acronym TEC had already been defined

l. 715: "Note added" looks like a draft

A table with acronyms would help
