# Supplementary material for: A biochemical mechanism for time-encoding memory formation within individual synapses of Purkinje cells
Source: PLoS One. 2021 May 7;16(5):e0251172. doi: 10.1371/journal.pone.0251172 (PMC8104431; doi:10.1371/journal.pone.0251172)
Supplement: Attachment — Submitted filename: Reviewer_comments_2ndround.pdf [file pone.0251172.s010.pdf]

The authors significantly improved the readability of the paper and addressed the previous comments. In general, some additional effort is required to contextualize the paper and the modelling work within theories of conditioned response learning, and to present the impacts of the current results (besides suggesting some possible new experiments to test model predictions).

Detailed comments are suggested below to further improve the manuscript.

l.106-121: this part on the alternative hypotheses should be summarized, going directly to the conclusion and citing the literature.

l.121-137: the correlation between the protein mechanisms and the need for 2 stimuli (one from PFs and the other from CFs), to learn the response is not clear to me from this paragraph; please, try to rephrase.

The paragraph "Training with different ISI duration and time-encoding protein complexes" could be summarized more, going directly to the point and summarizing reasoning steps.

l.266-272: this paragraph introduces synapse potentiation/depression comments that should be better moved to the Discussion.

l.305-307: this comment should go directly in the paragraph where you discuss this.

l.559: the reference of experimental study is missing; it should be clearly stated in each part which experimental studies you are referring to, in order to compare model results to measures.

Fig. 3: labels of bottom panel should be changed. Please, also change colors for better clarity. From the figure it also looks like the v has the same trend between short and long ISI. How long are the short and long ISI values should be reported (in a simulation protocol paragraph of the methods, where the tests done should be described). How were the values chosen? Is the short ISI lower than 100ms?

Fig. 5: $\beta = 6.7645$, is the model so sensitive to β values (4th decimal digits specified)

Fig. 8: it is not clear how MMPIP enters in the model

l.766: the PC spike suppression should be quantified (reporting e.g. pause duration or drop in firing rate), possibly comparing to experimental data from reference papers

Paragraph "Specific experimental options to test the proposed model" is too detailed and could be summarized in a couple of examples.

l.870: the success of the model should be based on the comparison against data, not on the comparison between minimal and comprehensive model.

l.952: this comment should be stressed more throughout the paper (also in the Introduction). You are focusing on one single neuron level mechanism, how this impacts in network behavior? It is probably the whole network giving rise to learning as a results of multiple mechanisms at different scales.

l.1013: is this part intended to start with "Note added"?

It is not clear to me how the proposed model takes into account the role of the signal pathway from climbing fibers carrying the US.

Minor comments:

l.64: I would change “with” to “within” as the Conditioned Response is anticipatory with respect to the second stimulus.

l.75: “decrease in cAMP concentration, [cAMP]” would be better.

l.187: “mechanims” typo

l.189: AC?

Table 1: missing parenthesis in second raw

Fig. 4: x axis label? Legend could be one for the 4 panels and include also filled and empty dots meaning.

Fig. 9: legends could be merged
